# Why context matters when changing the diet: A narrative review of placebo, nocebo, and psychosocial context effects and implications for outcome research and nutrition counselling

**DOI:** 10.3389/fnut.2022.937065

**Published:** 2022-10-28

**Authors:** Melanie Neumann, Markus Antonius Wirtz, Gabriele Lutz, Alina Ernesti, Friedrich Edelhäuser

**Affiliations:** ^1^Department of Medicine, Faculty of Health, Integrated Curriculum for Anthroposophic Medicine (ICURAM) and Institute of Integrative Medicine, Witten/Herdecke University, Witten, Germany; ^2^Research Methods in Health Sciences, University of Education, Freiburg, Germany; ^3^Department of Psychosomatic Medicine, Gemeinschaftskrankenhaus Herdecke, Herdecke, Germany; ^4^Department of Medicine, Faculty of Health, Witten/Herdecke University, Witten, Germany; ^5^Department of Psychology and Psychotherapy, Faculty of Health, Witten/Herdecke University, Witten, Germany

**Keywords:** placebo effect, nocebo effect, psychosocial context effects, diet change, nutrition research, nutrition counselling/therapy, narrative review

## Abstract

Placebo (PE) and nocebo effects (NE) have been subjects of systematic research in medicine and psychotherapy for many decades to distinguish between the (specific) pharmacological effect of medication and the (unspecific) effect of the context. Despite this significant research, the awareness, operationalisation, and reflection of the multiplicity of PE, NE, and psychosocial context effects (PSCE) is currently limited when researching outcomes of diet changes in studies without randomisation and placebo control. This neglection is critical as it could systematically influence outcomes by moderating and mediating them and thus reducing the validity and evidence base of these studies. Therefore, we performed a (non-systematic) narrative review (NR) on the following objectives: (1) present a concise overview about the relevance of PE, NE, and PSCE in medicine and nutrition research; (2) review the current state of research on reflecting context effects when studying diet changes; (3) provide useful theoretical foundations *via* consideration and integration of micro- and macro context effects; (4) operationalise as hypotheses the potential PE, NE, and PSCE which are specific for researching diet changes; and (5) derive their impact for future research as well as for nutrition counselling. The electronic search in this NR for objective (2) identified *N* = 5 publications and for objective (4) we found *N* = 61 articles retrieved in the first round of search, additional references were identified by a manual and snowball search among the cited references resulting finally in *N* = 37. This NR offers a synoptical basis to foster awareness and operationalisation of a variety of PE, NE, and PSCE. Interdisciplinary research teams should monitor these factors using, e.g., qualitative, mixed-method studies, process evaluation, item bank approaches, moderator and mediator analysis that might reveal substantially new insights, and outcomes of relevance to science and nutrition counselling. Nevertheless, the present NR has several limitations, especially as it is non-systematic, because it is a very heterogeneous field of research, in which the topic we are investigating is usually regarded as marginal and subordinate. Therefore, future research should conduct systematic reviews and particularly theory-based primary studies (experimental research) on hypotheses of PE, NE, and PSCE in outcome research in diet changes.

## 1. Content and methodological background

Nutrition involves a wide range of characteristics and references: in addition to its essential life and health supporting as well as preventive functions, it is an important constructive element in emotional, social, and cultural contexts. It is a topic of interest to science, economics, politics and the media, and for health-related and ethical-ecological reasons it plays a key role of increasing social and economic significance at local, national, and global levels. Accordingly, nutrition is embedded in a comprehensive and complex context and thus “… *not a simple entity, but something that is constructed, negotiated, socialized and* contextualized.” [(1), S.468]. Therefore, the focus of this narrative review is on the *micro and macro context* of nutrition, i.e., placebo (abbreviated as “PE”), nocebo (abbreviated as “NE”) and psychosocial context factors (abbreviated as “PSCE”) which up to now have received less attention in outcome research of diet changes, especially in studies where nor RCT or control is applicable (see section 1.2). “Diet change” in this connection describes a self- or externally induced change in one or several areas of daily food intake with the aim of physical and/or mental improvement (e.g., switch from omnivorous to vegan diet or to sugar-free food to become healthier or reduce symptoms of diseases or becoming more self-confident or getting praise from the environment when reducing weight). The frequent disregard of context factors in non-randomised observational studies must be critically assessed since they have the potential to systematically confound and moderate the outcomes of diet changes (2, 3), and thus to considerably reduce the validity of statements on the effectiveness of diet changes in the absence of randomisation and placebo control.

Against this background, and in view of the significance of PE, NE as well as PSCE in medicine and nutrition research (section 1.1), the empirically depictable gaps in nutrition research on PE, NE, and PSCE (section 1.2), and the theoretically verifiable significance of micro and macro context factors for the development of health and illness (section 1.3), this narrative review has the following two primary objectives:

(1) To formulate hypotheses on classical PE and NE (section 2) and PSCE (section 3) which may be caused by diet changes. This primary aim is not only another step to close the above-mentioned research gap (section 1.2), but it is also intended to increase the awareness on this topic and thereby stimulate theoretical and empirical discourse in outcome research on diet changes (where non-RCT or control is possible) and help to strengthen the evidence base in nutritional science. That is, outcomes of diet changes can be interpreted with more validity and a sounder evidence base *via* consideration and control of PE, NE, and PSCE that may arise independent of the effect of food constituents (section 4.1).
(2) To integrate the identified findings and hypotheses into the practice and research of nutrition counselling on diet changes. This will help to intensify intended effects on clients and preventively reduce undesired effects (section 4.2).

Apart from these two primary objectives, this narrative review may in general be helpful in studies on, for example, eggs, food fats, low/high carb, alcohol, tea/coffee, antioxidants, or cholesterol. Findings from such studies are frequently contradictory in terms of subjective efficiency and the health risks and potentials involved, and ultimately add to the confusion of consumers (1, 3–5). Another possible approach to interpret the variance of such study results may involve PE, NE, and PSCE which might have an impact on the outcomes of these types of food.

The narrative review style has been selected for this study, because we wish to give a first overview and impression of a complex and huge issue. Furthermore, PE, NE, and PSCE are usually not in the main focus of research study. These effects are often considered to be of secondary importance and are reflected on and discussed in connection with the reflection of the primary study results. Therefore, a systematic approach would have exceeded the content of one paper as we did address numerous hypotheses (compare section 2 and 3).

We did perform a literature search for the present study in autumn 2020 and again in January 2022. The electronic search included several databases, i.e., PubMed, PsycArticles, PsyJournals, Embase, Psychology and Behavioral Sciences Collection, PubPsych and PsyDok, and Google Scholar in combination with snowball literature review. The search terms “nutrition,” “diet,” “diet change,” “diet interventions,” “context effect/factor,” “placebo effect,” “nocebo effect,” “expectations,” and “psychosocial effects” were used for the literature review in section 1.2. For sections 2 and 3 we also used these search terms in combination with the key words of each topic of the hypotheses, and we separately searched the key words of these topics in the above-mentioned databases.

The inclusion criteria were: all types of articles, literature from textbooks, also grey literature, and related only to humans. The exclusion criteria were: articles for which full text was not available, were not in English or German.

For section 1.2 we found *N* = 5 publications and for sections 2 and 3 we identified *N* = 61 articles retrieved in the first round of search. Additional references were identified by a manual and snowball search among the cited references resulting finally in *N* = 37. Overall *N* = 54 references were excluded due to (a) no methods described, (b) importance of journal, (c) number of references referring in the article, and (d) content redundancy.

It should be noted that is not the purpose of this narrative review to present the history and comprehensive literature on PE, NE, and PSCE from the past decades, or to explain the detailed psycho-neurobiological mechanisms of PE and NE (6–10).

### 1.1 Relevance of placebo, nocebo, and psychosocial context effects in medicine and nutrition research

Placebo, NE, and PSCE are deemed to be part of daily patient care in medicine (11, 12) as well as in psychotherapy (13, 14) and play an important role in clinical research. For approximately 80 years they have therefore been a subject of systematic research mainly in medicine (6, 7), to distinguish between the (specific) pharmacological effect of a drug and the (non-specific) effect of the medication context [such as colour and size of a pill, physician’s behaviour (12, 15)]. Benedetti et al. (16) use a broader definition of the PE which they also call the “psychosocial context effect,” since a therapeutic impact on the patient arises from the entire context of an intervention.

According to an older definition by Shapiro (17), a placebo (“I will please”) is a sham medication without pharmacological substance. In connection with foodstuffs the terms placebo or nocebo are, strictly speaking, not correct; instead, we should speak of placebo- or nocebo-like factors because they contain demonstrable substances with assumably effective properties. A PE is only given when effects occur which are not immediately caused by ingredients.

Moreover, e.g., the German Medical Association [(11), p. 4] uses a broader concept of the PE: *“Treatment with an active drug also involves a placebo effect, modulated by the respective setting, patients’ and physicians’ expectations, and the degree of success in doctor-patient interaction.”* [cf. also: (12, 15)].

Nocebo effects (“I will harm”) are the opposite and defined as follows: “*With the placebo effect, positive expectations induced by interventions such as medication or surgical treatment have a positive influence on a patient’s course of illness, whereas with the nocebo effect, such interventions awaken or increase a patient’s fears that they make him or her ill.*” [(11), p. 9]. The risks and side effects of a drug described in great detail in the package leaflet, or medical counselling prior to surgery can have NE to which highly sensitive and depressive individuals are susceptible (11, 15, 18).

It should be noted that recent research does not address PE, NE, and PSCE as marginal or negligible or even as “merely” distorting or confounding the really interesting effects (19). This would imply the intention to systematically control or avoid them in studies. Following the reasoning of Benedetti et al. (16), patients perceive PSCE as therapeutically effective, that is, they are *per se* effective and substantially interact with primary effects (8–10, 20). In addition, PE and NE can be located physiologically and neuroanatomically at the brain level and have a somatic and psycho-neurobiological basis (6, 9, 10, 12, 15, 21–23). Miller and Kaptchuk [(24), p. 224] go one step further by saying: *“To promote a more accurate understanding of the elusive and confusing phenomenon known as the placebo effect, we suggest that it should be reconceptualized as ‘contextual healing.”’* PE, NE, and PSCE should therefore be consciously used in nutrition counselling and prevention to increase desired effects and reduce undesired effects (11, 14, 15).

**FIGURE 1:**
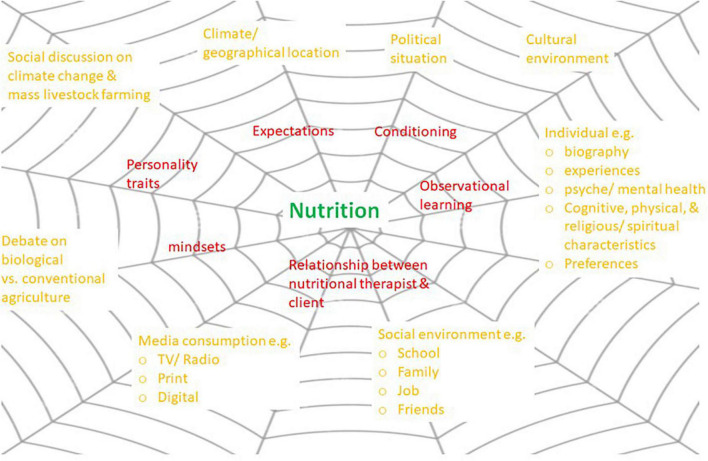
PE and NE (micro context, red) and PSCE (macro context, orange) in nutrition which interact with each other and have the potential to influence the outcomes of diet changes (source: own illustration).

Systematic theoretical reflection and empirical analysis of PE, NE, and PSCE – as in medicine – have not received much attention in research on diet changes so far (for details section 1.2), although they may occur in this area as well (25, 26). People consuming foodstuffs all have their individual biographies, experiences, preferences, and different cognitive, emotional-mental, physical, and religious/spiritual characteristics (27–29). Food consumption is further embedded in climatic-geographical, political, economic, cultural, and social contexts (30, 31). Due to a heightened public awareness of climate crisis, limited planetary resources/one health and mass livestock farming, a number of additional context factors have emerged over the past years in connection with an ecological and ethical-moral discussion right across society, including a debate on organic versus conventional farming (1, 32). Apart from such psychosocial context factors (macro context), the classical PE and NE (micro context) with their long history of intensive research in medicine and psychotherapy may also have a significant impact in nutrition and become PE, NE, and PSCE. In particular, these include expectations, conditioning, observational learning, personality traits, mindsets, and the relationship between therapist/physician and client (for details and hypotheses cf. section 2). Figure 1 gives a graphic illustration and synoptical overview of these potential factors from the micro and macro context with an impact on diet changes. The network structure in Figure 1 also reveals the interactions between these factors.

### 1.2 Reflection of context factors in the outcomes research of diet changes

As mentioned above, the micro and macro factors illustrated in Figure 1 have so far not been sufficiently described and pointed out, reflected, discussed, and systematically explored from an interdisciplinary perspective in nutritional research. Our narrative review identified only a small number of results: some publications are available from studies on the impact of expectations and (health-related) product information on the subjective perception of foodstuffs (33, 34) and in some cases their physiological effects (35, 36). A small number of placebo-controlled studies was found [for an overview see (3)] where study objectives and content permitted the use of placebos (37–40). But there is a lack of studies with operationalisation of and reflections on PE, NE, and PSCE and their systematic theoretical and empirical analysis. Approaches to this topic may only be found in parts in *N* = 5 publications, i.e., the position paper by (25) on “*The Total Food Effect: Exploring Placebo Analogies in Diet and Food Culture*” and partially also in (1–3, 41). However, compared to the present narrative review, these papers tend to give only a basic overview and no comprehensive summary of potential operationalised PE, NE, and PSCE (cf. sections 2 and 3) and their direct and indirect implications for outcome research on diet changes (without RCT or placebo control) and for nutritional counselling. Also, Mirmiran et al. (2), Staudacher et al. (26), and Yao et al. (42) do not explicitly focus on these issues in their papers; they address questions of methodology and options of placebo control in studies on diet changes. Only Costa et al. in a more recent qualitative study [(43), p. 1] suggest “… *that the healing potential of veganism, is derived from this passionate investment of the self that redefines young women’s ways of being in the world.”*

One possible explanation of the previously limited scientific investigation of PE, NE, and PSCE may be that – due to the object of investigation – it is often not feasible in nutritional research to conduct the gold standard of RCT (44), i.e., randomisation and placebo control (2, 26). A placebo condition may be much easier to implement in a drug trial whereby the active and placebo intervention is identical albeit for the active ingredient, whilst in dietary interventions this is more difficult to achieve particularly when working with whole foods. International guidelines on “Clinical trials in dietary interventions” are not yet available – in contrast to medicine (26, 42, 45). This problem is inherent to nutritional research (25, 42) and may be the reason why there is so little awareness of PE, NE, and PSCE. The *biomedical* research paradigm primarily applied in nutritional research might be another source of this lack of research or of guidelines. The following section therefore summarises two recognised models which illustrate the significance of the micro and macro context for the generation of health and illness and may thus serve to expand the research paradigm in nutritional sciences.

### 1.3 Models illustrating the influence of micro and macro context on health and illness

**FIGURE 2:**
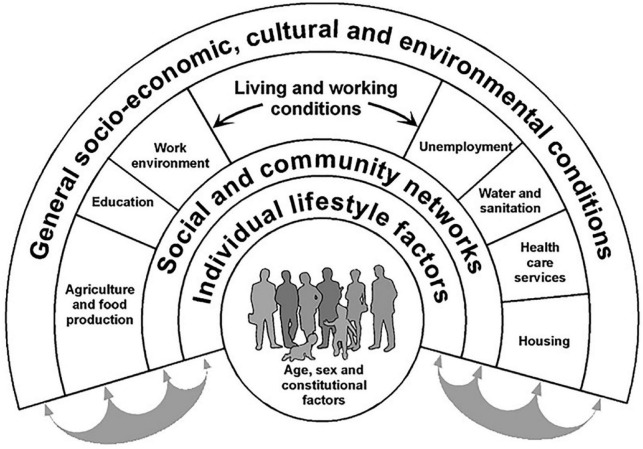
Dahlgren–Whitehead model of health determinants [from: (178), p. 22, (180), p. 36]. Source: adapted from Dahlgren and Whitehead (46).

Humans as consumers of food and as primarily responsible for their own health and illness are embedded in a comprehensive micro and macro context (cf. Figure 1). This fact is illustrated, among others, in the well-known “Dahlgren–Whitehead model of health determinants” (Figure 2; also known as “rainbow model”), originally developed by Dahlgren and Whitehead in 1991 and frequently referred to in research and health policy, for example, by the WHO (46) ever since. Based on their public health perspective, the authors describe context factors on the macro level which may have an impact on individual health. The hypothesis may be inferred from this model that the depicted macro context factors (Figure 2) can also be effective in studies on nutritional research and diet changes.

**FIGURE 3:**
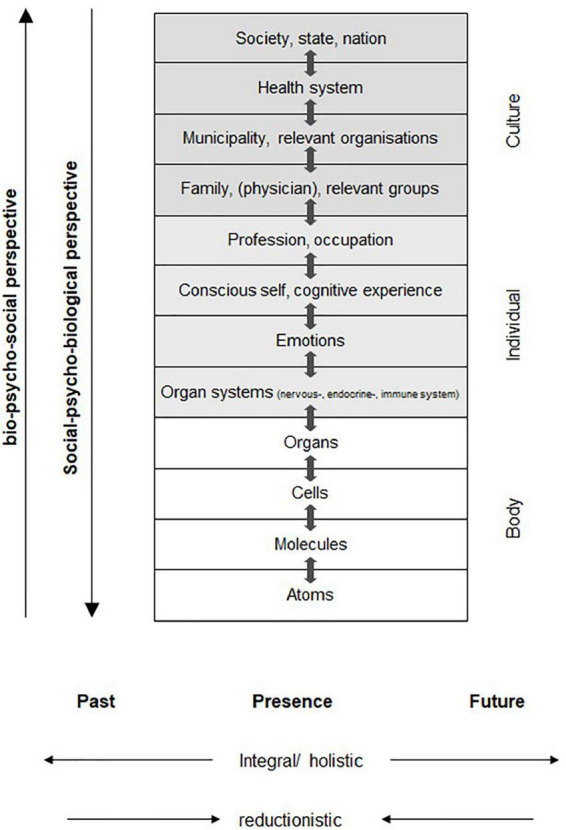
Biopsychosocial model of illness and health in line with (47, 179).

An essential part of theoretical foundation in psychosomatic medicine is the biopsychosocial model of illness and health (Figure 3) according to von Uexküll [(47); also cf. (48)]. It highlights the micro context of the development of health and by now has also become established and empirically validated in medicine (49); an overview of studies examining the model see (50) and psychoneuroimmunology (51, 52). It is occasionally referred to by nutritional researchers but receives little or no attention as a paradigm in research on diet changes [exceptions: (27, 53)]. But an isolated search on “physical,” “mental,” “social,” or “environmental” factors has proved to be not very helpful for an appropriate description and understanding of complex phenomena such as health and illness and the development of effective (nutritional) strategies for counselling and prevention. As a rule, all aspects are involved in each illness and also in the outcomes of diet changes (47, 54). According to the biopsychosocial model (Figure 3), the levels of “body,” “individual,” and “culture” and their respective sublevels interact with each other and thus contribute to health and illness (51, 52, 55).

Nutritional research might profit from these expansions of the biopsychosocial model by further micro and macro factors (cf. Figures 2, 3) as this would expand the research paradigm and thus serve to theoretically describe and systematically explore the impact of various context factors on, for example, the outcomes of diet changes, especially in studies where no RCT or control is possible. The following sections will describe details of this potential impact in the form of hypotheses.

## 2. Hypotheses on classical placebo and nocebo effects in diet changes

The hypotheses described in the subsequent sections are not exhaustive but focus on the operationalisation of the major classical placebo- and nocebo-like (section 2) as well as psychosocial context factors (section 3).

2.1 Diet changes are interventions which entail conscious and subconscious expectations and beliefs (56–58). Generally, we can motivate ourselves to changes especially if we expect an improvement of the existing situation, as illustrated by models such as the Health Belief Modell (59), the Transtheoretical Model (60), the Prototype/Willingness Model (61, 62), and the Health Action Process Approach (63). As a rule, individuals change their diet because they have expectations, e.g., health-promoting, weight-reducing effect, and/or appreciation from the social environment. In combination with the degree of suffering (e.g., caused by illness), the effects of expectation and belief will probably be even stronger (64), since illness tends to emphasize the meaning of and hope for possible external cures (here: diet change). All in all, expectations may have a considerable positive or negative influence on body and mind: “*The expectations of individuals are a powerful modulator of their cognitive, emotional and physical experiences.”* [(65), p. 3].
2.2 In societies dominated by the economy and the media, individuals are subject to influences from a wide range of information sources (TV, internet, film documentaries, social media), such as advertising of foods and supplements, the opinions of researchers and experts in nutrition, health or fitness, and have improved access to scientific studies (66, 67). In recent years, different forms of diet frequently were the subject of a – sometimes highly emotional – debate in public and digital media. Depending on type and setting, individuals are more or less susceptible to such influences. What we have here is model learning (68), and this media impact can induce many people to change their individual diet-related expectations, beliefs and attributions of meaning, and correspondingly may produce PE and NE (69).
2.3 Only approximately 4–6% of individuals change their diet on recommendation by nutritionists and nutritional physicians (70). One might therefore assume that the classical PE of the therapist–patient relation which is so influential, e.g., in medicine (21, 71–74), psychotherapy (13), physiotherapy (65), and naturopathy (75) does not occur with diet changes (25). But in the field of nutrition, we have manifold medial channels such as TV, radio and print media and in particular digital media (e.g., Internet, YouTube, Instagram, and Twitter) as extensive sources of not only information (76, 77) but also of influence [see section 2.2; (25)] and of peer pressure (59, 78–80), probably comparable to the influence of the above-mentioned therapist–patient relation. In addition, we have an individual’s (analogous) social environment of primarily family, relatives, friends, colleagues, fellow students whose nutritional behaviours and habits may also have an impact on one’s own diet and dietary changes (29, 81–83). Cooking and eating together is no longer just a daily necessity but increasingly an element of our social life and recreational activities (84), often combined with intensive communication on diets and diet change. This means that diet changes are highly influenced by role models and influencers from digital media and also from the (analogous) social environment; this process involves model learning and at the same time an impact on subjective expectations (68).
2.4 Mindsets, defined as “… *a broad set of viewpoints that compose one’s outlook on life”* [(65), p. 5], may also interact with PE and NE. Studies by Crum et al. (85, 86) documented that a targeted manipulation of optimism can be used to instigate positive change in subjective and objective parameters of health and wellbeing. In this manner, PE may occur in test persons who in studies on diet change or in nutritional counselling are consciously or unconsciously subjected to positive influences regarding a product and/or behaviour (e.g., with so-called “superfoods” where the name alone encourages optimism). On the other hand, negative expectations may also be triggered to cause NE.
2.5 Medical studies moreover document effects of personality traits, such as an optimistic or pessimistic personality. Nocebo reactions are more likely to occur in persons with high levels of trait and state anxiety [for on overview: (65)]. These and possibly other character traits [e.g., openness to new experiences, extraversion; (87)] may therefore cause PE and NE in diet changes. In addition, it appears plausible that mental vulnerabilities as for example high anxiety scores (88, 89) may lead to NE, especially in view of studies testing diet changes in persons with chronic diseases, allergies, or food intolerances (90). Anxiety may also play a role in decisions for or against food supplements or a diet change (e.g., vitamin B12 for vegans), in particular where children and adolescents or pregnant women are concerned.
2.6 Important learning processes in the context of PE and NE are classical and operant conditioning which may also become effective in diet changes [(12, 27), overview: (65, 91)]. Examples of classical conditioning are abdominal pain or nausea after consumption of a particular foodstuff. In other words, a neutral stimulus (such as fatty sauce) triggers a hitherto unconditioned reaction (like nausea) and finally turns into a conditioned stimulus which may trigger a conditioned reaction (example: the mere sight, smell and/or thought of fatty sauce causes nausea). In operant conditioning, certain behaviours are influenced by consequences following that behaviour immediately or with a delay. Positive consequences help to strengthen the respective behaviour in the sense of reinforcement (such as praise, recognition, encouragement to consume preferred foodstuffs or to avoid negatively assessed food) whereas unpleasant consequences have a punishing effect with resulting behaviour reduction [such as withdrawal of affection or disparaging comments if standards of the social environment are not met; (92)]. This means that, above all, reactions from the social environment in the sense of operant conditioning can have an impact on nutritional preferences, aversions, reactions (physical and psychological) and dietary habits and changes (93). These insights are already finding use in nutritional counselling (91). For research on diet changes this implies that test persons have already gone through certain conditioning processes which may have unconscious PE and NE.

In conclusion to section 2 it should be noted that comprehensive research was carried out on placebo response, i.e., on the question as to which individuals with which characteristics react to placebos [compare (94)]. Further discussion of the issue does, however, not appear to be appropriate in this context as it would exceed the scope of this narrative review.

## 3. Hypotheses on psychosocial context effects in diet changes

Apart from classical PE and NE described above, there is a range of other PSCE, such as psychological (section 3.1), health-psychological (section 3.2), and social (section 3.3), which may have a positive or negative impact on outcomes in diet change in uncontrolled and/or non-RCT studies.

### 3.1 Hypotheses on psychological context effects

3.1.1 Some degree of openness and, most importantly, motivation are basic requirements in any kind of behavioural change (95, 96). Possible stimuli are health problems, good perception of one’s body and its needs, external criticism etc. Motivation is an important basis to develop willpower (97) and determination, such as the will to become active or the will to give something up, which are also required to change one’s diet. After a successful diet change, motivation and willpower can be further strengthened with positive effects in other areas of life (e.g., career and exercise). In case of a failed diet change, the positive PE may turn into a NE (e.g., into reactance, frustration, and resignation) and spread to other areas of life. Another aspect to be studied is how an intrinsic versus extrinsic motivation (95, 97–99) moderates the effect of diet changes: it is argued that when individuals are intrinsically motivated it is more likely that long-term behaviour change occurs (100). Likewise, de Ridder et al. (101) found that people with a higher intrinsic motivation were more willing to implement a healthy dietary goal.
3.1.2 Chan et al. (102) found that self-efficacy is an important predictor of intentions to engage in healthy eating. In other words, a further important requirement for a diet change is a certain degree of self-efficacy (103), an individual’s answer to the question: *“What do I believe I am capable of doing?”* or *“Can I handle this diet change (mostly) on my own?”* Success probability, efficacy and sustainability increase with the confidence to cope with this new challenge (104), and the resulting improved self-efficacy will again have a positive influence on other spheres of life (e.g., work and leisure time) (105). In the “Process model of health behaviour” (106) self-efficacy supports the development of motivation and intention.
3.1.3 A diet change, especially at the beginning, demands that you take time to clarify various open questions, such as: Where to shop for the new ingredients, where to find new recipes, how to ensure provision with all nutrients and vitamins? The fact that you set aside time for yourself, and your health indicates that some pre-existing degree of self-esteem is required and helpful (107). A successful diet change can strengthen this self-esteem and may also result in more mindfulness and self-care. But gender-specific differences can be observed in the context of diet changes, whereby self-esteem scores are lower in women compared to men (108).
3.1.4 Diet changes, as most other changes in life, inevitably bring new positive and negative experiences. These may enhance an individual’s personal growth, for example, on emotional, social, physical, and behavioural levels (43). A plant-based diet change may give fresh impetus to sustainable behaviour (109) or improved body awareness (43). Negative experiences on the other hand can have a negative effect on self-development, such as condemnation of others, more stress and hassle from discussing this type of diet (110). Most of all, diet change can result in tension and stress, particularly at the beginning when many new aspects need to be considered and handled (e.g., shopping, cooking, information to and discussion with one’s social environment, and food supplements) (111). Depending on individual constitution and susceptibility, this stress may be perceived as challenging (in the sense of eustress) and by others as excessively demanding (in the sense of distress) (112). Eustress is what we need for personal development, but distress for long periods and in chronic form is a major risk factor for mental and physical illness, as confirmed by comprehensive research over the past decades (113–116). Permanent distress as a result of diet change, for whatever subjective reasons, in combination with worries or fears may turn a well-considered and healthy diet change into a “unconsciously unhealthy” behaviour change. One example would be somebody who has cognitively opted for a plant-based diet but ignores his or her individual and emotional needs and as a result is permanently stressed because he or she misses, e.g., the enjoyment of cheese, ice cream etc. But the exact opposite is also a possibility: a dedicated person rises to the challenge at various levels and thus gains resources for future challenges in life.

Forms of nutrition may also be associated with guilt or avoidance of guilt (117), especially a vegetarian or a vegan diet, but also the purchase of organic and fair-trade products and the refusal to eat or wear products from “animal children” (lambs and calves) (118). Buying regional products and avoiding ecologically questionable products (e.g., palm fat) or sweets and other luxury foods can be more or less conscious strategies to avoid feelings of guilt. Immediate results may be a clean conscience, relief from feelings of guilt, and more understanding and empathy, and therefore improved emotional wellbeing (e.g., prevention of depression). In the contrary case, guilty feelings can become even more pronounced. Chronic feelings of guilt may lead to despondency, resignation, poorer self-efficacy, and impaired emotional wellbeing. They may in addition increase the likelihood of psychiatric disorders like depression (119–121) and – in the sense of “embodiment” – cause a physical feeling of heaviness (122–124). If personal feelings of guilt are then projected on others in an attempt to convert them to forms of “guilt-free diet” as described above, this can have social implications as mentioned in hypothesis 3.3.1.

The self-determination theory (SDT) (95, 97–99) is a comprehensive so-called macro theory which describes the experience of autonomy, competence and relatedness as fundamental psychological needs in humans and essential predictors of the experience of self-determination and thus of intrinsic motivation. The extensive research on SDT^1^ has moreover shown that an individual’s psychological and physical wellbeing (95, 98), subjectively perceived vitality and energy levels (98, 125) as further important outcomes of diet change studies rise with the extent to which these basic needs are met. Transferring this proven and excellently researched psychological theory to the motivation and efficacy of changes in behaviour (126) and diet, we see that the experience of autonomy, competence and relatedness may also constitute necessary motivational and outcome-relevant preconditions of intervention research and/or counselling with an impact on nutrition and may even have reinforcing positive effects (97). If preconditions according to the SDT are not fulfilled this may cause NE: A diet change may for example be terminated quickly if it does not leave sufficient autonomy to somebody with a strong need for self-determination, or if a nutrition consultant makes a client feel uninformed, incapable and incompetent.

### 3.2 Hypotheses on health-psychological context factors

3.2.1 A diet change entails the need to seek more information (cf. hypothesis 3.1.3), and this research may yield additional health-related and health-promoting facts and/or methods, with an increased awareness of one’s own health and other spheres of life as a possible result. However, this positive PE can turn into a NE if the search for information becomes so obsessive that affected individuals have nothing but critical thoughts about many areas of life and become dispirited, or if they believe they need many food supplements and are concerned and worried about a possible lack of nutrients. Nearly every form of nutrition entails a theory of its own about efficacy which may raise new questions and considerations and thus can induce either positive reinforcement or uncertainty.
3.2.2 Every change in life presupposes some degree of self-control in the sense of a willingness to be disciplined and give something up (127–130). This also applies to dietary shifts which imply drastic changes that make themselves felt several times per day. A successful diet change can have a positive impact not only on self-efficacy and self-confidence but on other spheres of life as well, such as physical activity, job, and/or household. Avoidance of certain foodstuffs may also stimulate creativity because many dishes will now have to be prepared differently, e.g., without sugar, fat or gluten, which requires rethinking and reorganising. But rigid adherence to these virtues has a negative side as well: individuals with (tendencies to) eating disorders or obsessive-compulsive disorders (OCDs) may be primarily attracted by forms of diet change that require a high degree of discipline and renunciation (131, 132). Eating disorders or OCDs may be triggered or reinforced in this manner and lead to an NE.
3.2.3 Body awareness is a multifaceted concept that has been defined as attending to, and identifying the inner sensations and overall state of the body and its changes in response to emotional and environmental shifts (133, 134). In other words, a diet change may presuppose and/or result in improved body awareness. It can be a precondition because you may intuitively feel that your body needs a different form of nutrition for you to experience more energy and vitality. It can be a result if changes in food intake produce new body sensations (e.g., altered intestinal activity) or needs (e.g., the need for more exercise because the body has more energy) which must be perceived and addressed. Research in somatic theory confirms this hypothesis as it shows that body awareness is linked to the conscious internal processes of self-knowledge and regulation that facilitate human growth and wellbeing (135–138). Furthermore, it is obvious that Western society mainly relies on rationality, analysis, and reason as a basis for decisions, and that we tend to listen to expert opinion more than to our own intuition and body signals. This is why the decision to change one’s diet may have essentially cognitive reasons and hardly relation to personal needs or body perception. Possible risks involved in this case are, e.g., wrong nutrition and/or quick termination of a diet due to frustration or lack of success.

### 3.3 Hypotheses on social context factors

3.3.1 Like any other alteration in life, a deliberate and healthy diet change can stimulate reflection and constructive debate with the social environment, e.g., on nutrition and health, environment issues, sustainability. But the opposite is also possible, and destructive discussions may lead to social conflicts or even social exclusion. The social environment (e.g., colleagues at work, partners, and parents) can facilitate and support a conscious and healthy diet change (e.g., partner joins in) or obstruct it (e.g., parents prevent their child’s diet change) (139, 140). On the other hand, “diet changers” may come across as opinionated and dogmatic, or they show understanding and empathy for the “other” side, with corresponding positive or negative social effects on both sides (110). In general, research from recent decades (141) clearly shows that social support and social exclusion respectively can have tremendous positive and negative implications for psychological and physical wellbeing as well as for motivation (83).
3.3.2 Diet changes may also have an impact on behaviour regarding ecological sustainability behaviour and also on other spheres of society and life (142): one positive result may be the avoidance of products that are dubious or dangerous from the perspectives of ecology, animal welfare, or human rights (43). These include, for example: palm fat, animal products, products from poor labour conditions, and clothing produced with leather, fur or downy feathers. Other possible consequences are consumption of regenerative energies (e.g., green electricity), reduced energy consumption for household and/or mobility, more physical activities and sports, or more commitment to nature conservation. These wider effects can have a positive impact on mind (e.g., less guilty feelings and more self-efficacy) and body (e.g., more exercise and more sports). But overly rigid and strict adherence may impair not only one’s quality of life (e.g., total avoidance of products formerly relished, and therefore less enjoyment of life) but also the quality of social relations.
3.3.3 Today we know that the consumption of food contributes, among other things, to the construction of social identities and standards (143–147). Novel innovative diets and above all current trends (e.g., veganism, clean eating, raw fruit, and vegetables) can give confidence and assertiveness because you become part of a group or social movement where you not only feel at home, but also in a “community” and “on the right side” (110, 119, 148). This feeling of belonging to a group can stimulate personal growth and identity formation in distinguishing oneself from others (149, 150). Adversely, separation and being different from others may result in rejection and social conflict if the idea is to demand acceptance of one’s own differentness and not to tolerate that of others in return (110). Negative social effects may follow as described above in hypothesis 3.3.1.

## 4 Discussion

The hypotheses collated in sections 2 and 3 point to various PE, NE, and PSCE that may occur in connection with diet changes; subsequent desired and undesired effects can be identified in studies and in nutritional counselling. The following sections summarise (section 4.1) and discuss implications of these hypotheses for future outcome research on diet changes (section 4.2) as well as for the practice of nutrition counselling and research (section 4.3) ending with a conclusion (section 4.4) by providing take home messages (Table 1) and strength and limitations of this narrative review.

**TABLE 1:** Take home messages.

•	From a medical perspective, PE, NE, and PSCE have a psycho-neurobiological basis. These effects are determined by the entire context around a diet change, e.g., by the patient and his/her environment, the type of diet change, the setting, the nutrition counsellor.
•	From a theoretical health perspective, the “Dahlgren–Whitehead model of health determinants” and the “Biopsychosocial model of illness and health” indicate the significance of context factors for the generation of health and illness and may thus serve to expand the biomedical research paradigm currently prevalent in nutritional sciences.
•	To distinguish between the (specific) treatment effect of a diet change (i.e., the ingredients) and the (non-specific) context effects as well as the according interaction effects, the awareness, operationalisation, systematic theoretical reflection and research of PE, NE, and PSCE is currently deficient and needs more attention, especially in studies on diet changes where no RCT or control is possible.
•	Analysing moderating and mediating effects of PE, NE, and PSCE may help to understand and model the causal pathways and impacts of diet chance on the outcomes more comprehensively and validly.
•	Classical PE and NE are hypothesised to influence studies on diet changes including, e.g., diet-related expectations and beliefs, model learning, mindsets, personality traits/mental vulnerabilities, and (classical/operant) conditioning.
•	Psychological PE and NE are postulated as well to have an impact when researching diet changes involving, e.g., (intrinsic/extrinsic) motivation, self-efficacy and -esteem, positive/negative experiences with the diet change, guilt/avoidance of guilt as well as autonomy, competency, and relatedness as the three fundamental psychological needs.
•	Health-psychological PE and NE are also assumed to impact outcomes of diet changes, e.g., an increased awareness towards health-related and -promoting facts/methods due to diet change, the willingness to be disciplined and abandon undesirable behaviours, as well as body awareness.
•	Social PE and NE are hypothesised to affect outcomes of diet changes, e.g., *via* social support and exclusion, ecological sustainability behaviour, and group/social movements.
•	Recommendations for non-RCT/uncontrolled studies are to monitor the hypothesised PE, NE, and PSCE before, during and after studying diet changes *via* qualitative, mixed-method studies, process evaluation, item bank approaches, moderator and mediator analysis, experimental manipulation in primary studies and drawing up and international consensus statement on it.
•	PE, NE, and PSCE in diet changes are also professional topics that should be integrated in nutrition counselling education as well as in daily practice. Applying single-case research designs may help to account for these context factors when investigating individual-based counselling processes.

### 4.1 Synopsis and general methodological reflection

**FIGURE 4:**
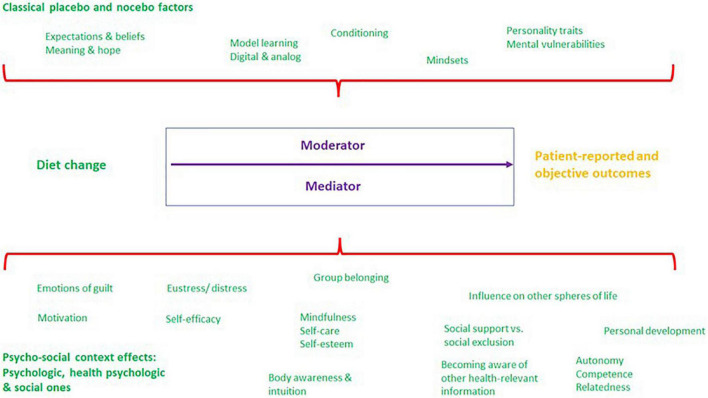
Classical placebo- and nocebo-like as well as further psychosocial context factors acting as moderator and/or mediator variables (definition see below in section 4.1) that may have a positive and/or negative impact on patient-reported and objective outcomes in diet changes (source: own illustration).

Figure 4 summarises the hypotheses described in sections 2 and 3 to facilitate the subsequent discussion.

The hypotheses described and operationalised for the first time in this form in sections 2 and 3 illustrate the various factors which may have a positive and/or negative impact on the material effectivity of nutrition (specific effect), as effect mechanisms in the sense of a “psychosocial context effect” (10, 16). This variety of analysable factors underlines the actual significance of the models of micro and macro context presented earlier (cf. section 1.3) in research on diet changes on the one hand. On the other hand, the large number of context factors calls attention to the complexity and the many interactions that may occur in the context of non-RCT and uncontrolled outcome studies on diet changes (Figure 4).

Apart from knowledge of the range of effect mechanisms in diet changes described above, scientific studies also need to take selection mechanisms into account. The hypotheses suggest that every diet change implies a pre-existing degree of health-promoting attitude and capacity. Individuals in better mental, cognitive and physical health are generally more likely to change their diet (151–153). This means that those who plan to switch to a healthier form of nutrition often have some degree of health-promoting resources enabling them to initiate a diet change with consciousness and autonomy.

Awareness and knowledge of effect and selection mechanisms is of specific importance in nutrition research, in particular to develop dietary recommendations for the population of a country or for people with (chronic) diseases. Our hypotheses (sections 2 and 3) indicate that – notwithstanding the positive intention – a diet change may also have negative psychological and (as a result) physical effects (nocebo effects). This means that a diet which is healthy from a material/physiological perspective may involve risks and side effects depending on individual constitution; frequently these remain insufficiently explored and communicated. On the other hand, hypotheses described in section 3 point to a considerable psychosocial and probably also psychosomatic impact and chance potential in case of a successful diet change. However, the above-mentioned probable “positive selection bias” suggests that primarily people with better psychosocial and health-promoting resources will be able to seize their chance. This should be considered from the perspectives of science, health politics, and nutrition counselling.

### 4.2 Recommendations for outcome research on diet changes in non-RCT and uncontrolled studies

As initially mentioned (section 1.2), Mirmiran et al. (2), Staudacher et al. (26), and Yao et al. (42) provide methodologies and options of placebo control in studies on diet changes. However, the challenge for outcome research where a RCT or placebo control cannot be applied, is to methodically monitor the PE, NE, and PSCE described in the above theses. Ideally, monitoring should be performed prior to, during and upon completion of studies on diet changes for more valid and evidence-based conclusions to be made (154–158). The way in which a methodical check of PE, NE, and PSCE can be performed without RCT is also very important in order to pay due attention to the above-mentioned complexity of interactions, possible domino effects and a processual experience of dietary change. The context factors described in sections 2 and 3 should therefore be analysed not only with quantitative but also qualitative methods in the sense of a mixed method approach (159, 160) since qualitative and quantitative methods can be integrated to complement each other (154, 161–164). Qualitative studies [e.g., one-to-one interviews, group discussions, diaries, observation, and first-person research; (165)] and mixed method studies [e.g., qualitative preliminary studies, concomitant interviews, group discussions on how to interpret data from RCTs; (161)] place a stronger focus on the subjective patient perspective. Such a process evaluation [for details see MRC guideline (154)] is required to analyse the factors illustrated in Figure 4. Process evaluations serve to explore conditions, circumstances, and processes of mainly complex interventions and their implementation (154). Diet changes are among the so-called “complex interventions” (2) where concomitant qualitative methods are required for an in-depth understanding of complicated inner psychological and social correlations and of subjective alterations in condition and behaviour (154). Studies of this type should be embedded theoretically in a theory-based research paradigm as described in section 1.3, and accordingly be conducted by an interdisciplinary team of researchers.

An additional and economic methodological monitoring in studies on diet changes would be the idea of a item bank of contextual factors [compare (65)] which can be represented in a questionnaire that classifies patients/clients based on their personally and subjectively experienced contextual factors. Moreover, such a questionnaire can help nutrition counsellors to enrich and to intensify their counselling and to avoid nocebo effects.

One question to be primarily addressed in process evaluations is whether the factors described in the hypotheses have the effect of moderator or mediator variables (166) on the analysed patient-reported and objective outcome parameters and thus may influence or even induce the material or physiological (specific) effect of a diet change (cf. Figure 4). In case of a moderation, a third variable (here: PE, NE, and PSCE) influences the degree of correlation between a dependent (here: patient-reported and objective outcomes) and independent variable (here: diet change). In this manner, a moderator acts much like a garden sprinkler which you turn up or down to regulate the intensity of a relation. In contrast, a mediator mediates the relation between dependent and independent variables and makes the existence of the relation possible in the first place (166). In other words, without the mediator variable there would be no effect relationship between a diet change and a specific patient-reported or objective outcome. Moderator and mediator analyses thus appear not only to be essential to obtain evidence-based and more valid conclusions about certain diet changes and to identify desired and undesired effects, but also to investigate interactions of context factors (section 4.1). These analyses, as suggested earlier (section 1), might also provide more clarity about contradictory study results in many areas of efficacy research (e.g., on the influence of nutrition components such as eggs, fats, low/high carb, alcohol, coffee/tea, antioxidants, and cholesterol).

With an enlarged awareness and understanding of contextual factors, the scientific community can measure and analyse the impact of diet changes with greater precision in primary studies. That is, beyond the above-mentioned monitoring techniques, experimental manipulation of PE, NE, and PSCE is also recommended to examine their impact on patient outcomes as part of a systematic research, like in medicine (section 1.1). Ideally, primary studies should follow a theory-based research agenda aimed to assess the effects of contextual factors on various patient outcomes. Applying RCTs there is needed to compare a neutral diet change with an enriched or manipulated context (e.g., studying a vegan diet change *via* randomising study participants into diverse conditions, i.e., inducing positive/negative/no expectations, and in a next step *via* different channels of expectations, i.e., physician, digital). Furthermore, effects of influential conditions or diet programmes may be mediated or moderated by PE, NE, and PSCE. Only if these effects are systematically considered in controlled study designs, the actual impact conditions and impact processes can be identified validly.

Nevertheless, interdisciplinary and international collaboration to draw up a consensus statement, e.g., *via* round-table discussion and/or Delphi method, could be helpful not only to elucidate the role of PE, NE, and PSCE in nutrition research but also to agree on suitable methods to deal with them [cf. consensus statements, e.g., (14, 167)].

### 4.3 Recommendations for nutritional counselling and its research

Current nutritional counselling will profit in practice as well as in training and further education from an integration of effect and selection mechanisms, chances and risks of PE, NE, and PSCE as described above (section 4.1). An essential prerequisite for this, however, is an approach aligned with the models of micro and macro context described earlier (section 1.3), since nutritional science and counselling still appear to be dominated by the biomedical model. Changes in practice and in basic/further training will be very difficult without a shift in mindset but might be stimulated by intensified research into context factors.

The key focus in practical nutritional counselling, on ethical grounds (18, 168), is on how to maximise desired effects described in the hypotheses (sections 2 and 3) and to minimise or prevent undesired effects. This is even more relevant since the therapist–client relationship has a strong impact on health-related outcomes of patients in medicine, psychotherapy, and other therapies (e.g., physiotherapy) (cf. hypothesis 2.3 in section 2) and this applies equally to nutritional counselling. Context factors act as a continuous outcome-relevant influence throughout the entire counselling process, that is, during anamnesis, diagnosis, communication of prospects of a diet change, implementation advice, and the final success evaluation. In general, a positively acting context for nutritional counselling may be created *via*

○ Patient-oriented information [e.g., (169)],
○ An empathic (71, 73) attitude/behaviour on the part of the therapist that inspires confidence (11),
○ Empowerment in support of self-efficacy and individual responsibility,
○ Communication of positive expectations for a successful diet change, and
○ An authentic interpersonal relationship (72).

It appears reasonable to test the application of the above-described hypotheses (cf. sections 2 and 3) in daily practice of nutritional counselling under scientific investigation (e.g., in a first step with the above-mentioned item bank before, during and after a counselling process, section 4.2) with the idea being to translate them into “psychosocial communication recommendations for practicians” and into a curriculum in “psychosocial communication competences in the training and further education of nutritional therapists and physicians.” The latter would be of particular importance, considering that current curricula and textbooks on nutritional sciences (170), counselling (171) and medicine (172) do not devote much attention to these issues.

Finally, to investigate individual-based counselling processes in clients aiming to change their diet/eating habits we suggest to apply the recommendations in section 4.2. Additionally, intervention-based approaches should be adopted. There, different forms of PE-support and NE-avoidance can be applied and employed time-shifted. Thus, in different study participants (single-case research design) different series of counselling processes can be implemented und compared regarding the course of time (173–176). Additionally, the significance of PE, NE, and PSCE may contribute to enhance the conceptual foundation and contents of helpful counselling processes. Developing and evaluating appropriately enriched counselling concepts and counselling elements also offers an interesting desideratum for empirical research.

### 4.4 Conclusion: Strength and limitations of the present study

In summary (Table 1), this narrative review offers a synoptical basis for reflection and discussion on a variety of PE, NE, and PSCE in diet changes that are of relevance to outcome research and counselling, especially in studies where no RCTs or control are possible. Interdisciplinary research teams should systematically investigate the effectiveness and selection effects of these factors *via* the recommended methodological approaches (sections 4.1–4.3 and summary in Table 1) that might reveal substantially new insights and outcomes of relevance to science and counselling. In this manner they might be able to define in more detail not only the (specific) physiological effects of diet changes but also desired and undesired (non-specific) effects that are of practical significance in nutritional counselling. Analysing moderating and mediating effect of PE, NE. and PSCE which influence and characterise the effects from diet changes to nutrition outcomes may enlighten and deepen the understanding of underlying causal process models (177). The exploration of context factors in diet changes is still in the early stages and therefore constitutes a newly evolving and innovative field of research.

Beyond these strengths, our narrative review suffers from several limitations, mainly as it is non-systematic. Further limitations of a narrative review are that the nature of the method may be too subjective in the determination of which studies to include, the way the studies are analysed, and the conclusions drawn. Moreover, the possibility of misleading in drawing conclusions prevails and also the problem in determining and integrating complex interactions (that may exist) when a large set of studies is involved. Therefore, future research should conduct systematic reviews and particularly theory-based primary studies (experimental research, see section 4.2) on hypotheses of PE, NE, and PSCE in outcome research in diet changes. However, because the importance of PE, NE, and PSCE has often not been adequately addressed in research on diet changes to date, more (intervention) studies need to be conducted to provide sufficient substance for a systematic review.

## Author contributions

MN and FE: conceptualisation. MN and MW: literature research, methodology, and writing—original draft preparation. MN, MW, and FE: interpretation of reported studies and funding acquisition. MN: figures preparation. FE, GL, and AE: writing—review and revising. GL and AE: writing—editing. All authors gave final approval for all aspects of the work, agreed to be fully accountable for ensuring the integrity and accuracy of the work, and read and approved the final manuscript.

## Footnotes

1
https://selfdeterminationtheory.org/research/
